# Supplementary material for: The Brain in Context: A Scoping Review and Concept Definition of Neuro-Informed Policy and Practice
Source: Brain Sci. 2024 Dec 11;14(12):1243. doi: 10.3390/brainsci14121243 (PMC11674288; doi:10.3390/brainsci14121243)
Supplement: Supplementary file 1 [file brainsci-14-01243-s001.zip › NeuroInformedScopingReview_SupplementaryTableS1.pdf]

Supplementary Table S1

Overview of Publications Included in the Scoping Review

No^	Author	Date	Pub Type*	Location	Target Area(s)**	Focus+	Critique	Definition of NPP++
1	Andrews	2016	BC	USA	Ed	Pr	N	Y
2	Bakar & Ghani	2022	JA	Malaysia	Ed	Pr	N	Y
3	Bamber	2019	JA	UK	Ed	Pr	N	NE
4	Beeson & Field	2017	JA	USA	H	Pr	N	Y
5	Beeson & Miller	2019	JA	USA	H	Pr	N	Y
6	Billington	2017	JA	UK	Ed	Po, Pr	Y	NE
7	Blackburn	2009	D	USA	Ed	Pr	N	NE
8	Brewer & Murphy	2021	BC	Australia	H	Pr	N	NE
9	Busso & Pollack	2015	JA	USA	Ed	Po, Pr	Y	Y
10	Buzick	2020	D	USA	Ed	Pr	N	NE
11	Caine	2008	BC	USA	Ed	Pr	N	NE
12	Camargo et al.	2020	JA	UK	PE	Po, Pr	N	Y
13	CDC Harvard	2010	R	USA	Ed, SS, H	Po, Pr	N	NE
14	CDC Harvard	2016a	R	USA	SS	Po, Pr	N	NE
15	CDC Harvard	2016b	R	USA	Ed, SS, H	Po, Pr	N	NE
16	Chang et al.	2021	JA	USA	Ed	Pr	N	NE
17	Chapko	2015	R	USA/ UK	Ed, SS, H	Po, Pr	N	NE
18	Clement & Lovat	2012	JA	Australia	Ed	Po, Pr	Y	NE
19	Connell	2006	D	USA	Ed	Po, Pr	Y	NE
20	Cormier	2021	D	USA	Ed	Pr	Y	Y
21	Cuartas et al.	2022	JA	Colombia	SS	Pr	N	NE
22	De Bellis & Zisk	2014	JA	USA	H	Pr	N	NE
23	de Kogel et al.	2014	JA	Netherlands	L	Pr	Y	NE
24	de Nooyer & Lingard	2017	JA	Australia	Ed, H	Pr	N	NE
25	Deans & Larsen	2022	JA	Australia	Ed	Pr	N	Y
26	Di Gesu	2014	CP	Italy	Ed	Pr	N	Y
27	DiPietro	2000	JA	USA	SS	Po, Pr	Y	NE
28	Dow	2008	BC	USA	H	Pr	N	NE
29	Dubinsky	2010	JA	USA	Ed	Pr	Y	NE
30	Edelenbosch et al.	2015	JA	Netherlands	Ed	Pr	Y	NE
31	Ekhtiari, et al.	2017	BC	USA/Iran	H	Pr	Y	Y
32	Engle & Huffman	2010	JA	USA	H	Po, Pr	N	NE
33	Farah	2018	JA	USA	SS	Po	N	NE
34	Farrugia & Fraser	2017	JA	Australia	H	Po, Pr	Y	NE
35	Field et al.	2015	JA	USA	H	Pr	N	Y
36	Field et al.	2016	JA	USA	H	Pr	N	Y
37	Field et al.	2017	JA	USA	H	Pr	N	NE
38	Fishbane	2016	BC	USA	H	Pr	N	Y
39	Fisher et al.	2016	JA	USA	H	Pr	N	NE
40	Ford	2015	JA	USA	H	Pr	N	Y
41	Frederickson et al.	2013	JA	UK	Ed	Pr	Y	NE

No^	Author	Date	Pub Type*	Location	Target Area(s)**	Focus+	Critique	Definition of NPP++
42	Friedman	2006	R	USA	SS	Po	N	NE
43	Geake	2011	JA	Australia	Ed	Po, Pr	Y	Y
44	Goodwin	2018	R	USA	Ed	Pr	N	NE
45	Greenberg et al.	2004	BC	USA	Ed	Pr	N	NE
46	Gunnar	2006	I	USA	SS	Po	N	NE
47	Hardiman	2012	JA	USA	Ed	Pr	N	NE
48	Hardiman et al.	2012	JA	USA	Ed	Pr	Y	Y
49	Hohnen	2017	BC	UK	Ed	Po, Pr	Y	Y
50	Husak & Murphy	2013	BC	Australia	L	Pr	N	NE
51	Iacona & Johnson	2018	JA	USA	H	Pr	N	NE
52	Iyengar et al.	2021	R	USA	L	Po	N	NE
53	JohnBull & Hardiman	2023	JA	USA	Ed	Pr	N	Y
54	Kim & Zalaquett	2019	JA	USA	Ed, H	Pr	Y	Y
55	King et al.	2019	JA	USA	H	Pr	N	Y
56	Liu & Fisher	2022	JA	USA	H	Po	N	Y
57	Logue	2000	JA	USA	Ed	Pr	N	NE
58	Long et al.	2022	JA	Australia	H	Pr	N	NE
59	Lowe et al.	2015	JA	UK	SS	Po	Y	NE
60	Lown	2016	JA	USA	Ed, H	Pr	N	NE
61	Lucero	2018	JA	USA	SS, H	Pr	N	NE
62	Luke	2019	JA	USA	H	Pr	Y	NE
63	Luke et al.	2020	JA	USA	H	Pr	Y	Y
64	Mason et al.	2020	JA	USA	SS	Pr	N	NE
65	Matsumoto et al.	2020	JA	Japan	Ed	Pr	N	NE
66	McEwen	2017	JA	USA	H	Pr	N	NE
67	Medalia & Bellucci	2012	BC	USA	H	Pr	N	NE
68	Millei & Joronen	2016	JA	Australia	Ed	Po	Y	NE
69	Miller	2016	JA	USA	H	Pr	N	Y
70	Moore & Lagasse	2018	JA	USA	H	Pr	N	Y
71	NSCDC	2004a	WP	USA	Ed, SS, H	Po, Pr	N	NE
72	NSCDC	2004b	WP	USA	Ed, SS, H	Po, Pr	N	NE
73	NSCDC	2005	JA	USA	Ed, SS, H	Po, Pr	N	NE
74	NSCDC	2007a	R	USA	Ed, SS, H	Po, Pr	N	NE
75	NSCDC	2007b	WP	USA	Ed, SS, H	Po, Pr	N	NE
76	NSCDC	2014	R	USA	Ed, SS, H	Po, Pr	N	NE
77	Navalta et al.	2018	JA	USA	H	Pr	N	Y
78	Noble et al.	2017	JA	China	Ed, SS	Po	N	Y
79	OECD	2007	R	France	Ed	Pr	N	NE
80	Parris	2008	BC	USA	Ed	Pr	N	NE
81	Perry	2014	BC	USA	H	Pr	N	NE
82	Perry & Dobson	2013	JA	USA	H	Pr	N	NE
83	Petrocchi et al.	2022	BC	Italy	H	Pr	N	NE
84	Prendiville & Howard	2017	BC	Ireland	H	Pr	N	NE
85	Pykett	2019	BC	UK	SS	Po	Y	NE
86	Rauch & Mclean	2021	BC	USA	H	Pr	N	NE
87	Ray et al.	2023	JA	USA	H	Pr	N	NE

No^	Author	Date	Pub Type*	Location	Target Area(s)**	Focus+	Critique	Definition of NPP++
88	Rezapour, Assari et al.	2021	BC	Iran	Ed	Pr	N	NE
89	Rezapour et al.	2020	BC	Iran	H	Pr	N	NE
90	Rezapour, Barzegari et al.	2021	JA	Iran	H	Pr	N	NE
91	Russell-Chapin	2016	JA	USA	H	Pr	N	Y
92	Russell-Chapin et al.	2017	BC	USA	H	Pr	N	NE
93	Sanabria	2020	BC	USA	H	Pr	N	Y
94	Schildkrout	2016	JA	USA	H	Pr	N	Y
95	Schmidt et al.	2011	JA	UK	H	Pr	N	NE
96	Shi & Blau	2020	BC	USA	Ed	Pr	N	Y
97	Shonkoff	2011	JA	USA	Ed	Po, Pr	Y	NE
98	Shonkoff	2012	JA	USA	Ed, SS, H	Po	N	NE
99	Shonkoff	2014	JA	USA	Ed, SS, H	Po, Pr	N	NE
100	Shonkoff & Bales	2011	JA	USA	SS	Po	N	Y
101	Shonkoff & Fisher	2013	JA	USA	SS	Po, Pr	N	NE
102	Shonkoff & Garner	2012	R	USA	Ed, SS, H, PE	Po, Pr	N	NE
103	Sigman et al.	2014	JA	Argentina	Ed	Po, Pr	N	NE
104	Sinnamon	2019	BC	Australia	SS, H	Pr	N	NE
105	Skodje-Mack	2022	D	USA	H	Pr	N	Y
106	Stojek et al.	2018	JA	USA	H	Pr	N	NE
107	Takizawa et al.	2022	JA	Japan	H	Pr	N	NE
108	Thomas et al.	2019	JA	UK	Ed	Po, Pr	Y	Y
109	Verdejo-Garcia, Garcia-Fernandez et al.	2019	JA	Australia	H	Pr	N	NE
110	Verdejo-Garcia, Lorenzetti et al.	2019	JA	Australia	H	Po, Pr	N	NE
111	Vinke	2022	JA	Netherlands	H	Pr	N	NE
112	Vinogradov et al.	2021	BC	USA	H	Pr	N	Y
113	Wakefield & McPherson	2021	JA	USA	L	Pr	Y	NE
114	Watts-English et al.	2006	JA	USA	Ed, SS	Po, Pr	N	NE
115	Weatherston & Rosenblum	2018	BC	USA	SS	Po	N	NE
116	Zaleski et al.	2016	JA	USA	H	Pr	N	NE

Note. NSCDC=National Scientific Council on the Developing Child. CDC Harvard=Center on the Developing Child at Harvard University.
^ No=Source Number. These refer to the number given to each source in the Supplementary Tables Reference List.
* Publication type: R=Report; WP=Working paper; D=Dissertation; JA=Journal article; BC=Book chapter; I=Interview; CP=Conference paper
** Target Area: Ed=Education; SS=Social Services; H=Health; PE=Physical environments; L=Law
+ Focus: Po=Policy; Pr=Practice
++ Definition: Y=Yes, clear definition provided; NE=Not explicit, no explicit/clear definition provided
