# Supplementary material for: The Brain in Context: A Scoping Review and Concept Definition of Neuro-Informed Policy and Practice
Source: Brain Sci. 2024 Dec 11;14(12):1243. doi: 10.3390/brainsci14121243 (PMC11674288; doi:10.3390/brainsci14121243)
Supplement: Supplementary file 1 [file brainsci-14-01243-s001.zip › NeuroInformedScopingReview_SupplementaryTableS2.pdf]

Supplementary Table S2

Twelve Key NPP Knowledge Bases and Examples from relevant Publications

Key Knowledge Bases	Examples of Knowledge Bases
Brain Development	– The basic architecture of the brain is constructed through an ongoing process that begins before birth and continues into adulthood (15,68,74,75)<br>– The interactive influences of genes and experiences shape the architecture of the developing brain (15,17,58,74,75)<br>– Epigenetics (16,33,61,64,113)<br>– Prenatal brain development (33,97)<br>– Early phases of brain development (15,42,78,115)<br>– Adolescent brain development (34,80,113)
Brain Architecture	– The brain's many functions operate in a richly coordinated fashion with multiple systems throughout the body (15,93,115)<br>– Foundations of brain architecture are constructed early in life (15,42,68,71,74,115)<br>– Physiological arousal (36,66,84)
Brain Functions and Systems	– Executive functioning, including problem-solving, flexibility, planning, working memory, attention, inhibition, self-monitoring, self-regulation, and initiation (13,67,98,108,115)<br>– Neurobiology of thinking, emotions, and memory (2,3,4,7,11,16, 43,44,67,69,80,96)<br>– Perceptions and social cognition (2,108,112)<br>– Neuroscience behind language learning, literacy, and numeracy (2,3,7,11,47,49,79,96)
Neuroplasticity	– Neuroplasticity [brain reorganization] (42,66,69,75,98,105)<br>– Brain building continues through life (15,58,74)<br>– The brain sculpts itself based on interactions in the environment (7,42,68,71,74,115)
Individual Difference	– Developmental variability between people (11,79)<br>– Neural variability between individuals (11,52,79,95)<br>– Understanding of disability (79)
Sensitive Periods	– Importance of the early years in shaping the architecture of the brain (17,32,46,75)<br>– The developing brain is most malleable in the first few years (42,57,58,72,75,97,115)<br>– The importance of the first 1,000 days of life (72,78,115)

Key Knowledge Bases	Examples of Knowledge Bases
Sensitive Periods (cont.)	– During sensitive periods of early growth and development the evolving architecture of the brain (as well as the maturation of other organ systems) is highly receptive to a wide range of environmental signals or cues, whether positive or negative (13,17,32,46,75)
Stress	– Stress (excessive activation of stress response system) can affect emotions, reactions, and thoughts (14,98,107)<br>– Scientific understanding of how children cope with stress (73, 99)<br>– Types of stress and the effect on the developing brain (17,51,73, 97,114)<br>– Threat effects learning (1,3,15)<br>– The effects of toxic stress on brain development and behavior (15,46,73,76,97,98,107)<br>– Buffering of stress (4,24,25,88)
Brain-Body Connections	– Nutrition (17,32,46,91,103)<br>– Sleep (13,29,91,92,103)<br>– Physical activity (1,91,103)<br>– Stress and physical health (66,115)<br>– Effects of drugs on brain function and addiction (16,50,87,89, 110)
Resilience	– Nature, timing, and intensity of developmental adversities, and counter-balancing relationship, community, or cultural 'buffers' (14,24,33,58,64,73)<br>– Positive experiences (protective factors) help the brain grow and thrive across the life-course (13,14,17,24)<br>– Negative experiences (risk factors) hurt brain development and functioning (1,13,17,114)
Positive Relationships	– Children develop within an environment of relationships (15,28, 71)<br>– The importance of providing stable, supportive, and responsive relationships with caring adults (13,14,28,42,46,72,73,76,111)<br>– Serve and return interactions (71,74,115)
Trauma & Adversity	– ACEs (Adverse Childhood Experiences), including maltreatment, trauma, neglect, deprivation, violence, inconsistent care, malnutrition, and abuse (1,17,22,32,33,51,56, 61,72,77,99,104,111,114)<br>– The effects of adversity, including those associated with poverty, on children's development (17,22,73,76,78,98,104,114,115)<br>– Early relational adversity (115)<br>– The effects of trauma (10,51,61,64,65,66,76,81,104,116)

Key Knowledge Bases	Examples of Knowledge Bases
The Whole Person	– Biopsychosocial influences (9,10,77,83,95)<br>– The need for holistic approaches (22,77,79,93)<br>– Early childhood socio-economic circumstances are associated with brain functions across the lifespan (17,27,32,33,56,73,97, 98)<br>– Physical environments (12,27,33,47,83,102)

Note. Reference numbers refer to the number given to each source in the Supplementary Tables

Reference list.
