# Supplementary material for: The Brain in Context: A Scoping Review and Concept Definition of Neuro-Informed Policy and Practice
Source: Brain Sci. 2024 Dec 11;14(12):1243. doi: 10.3390/brainsci14121243 (PMC11674288; doi:10.3390/brainsci14121243)
Supplement: Supplementary file 1 [file brainsci-14-01243-s001.zip › NeuroInformedScopingReview_SupplementaryTablesReferenceList.pdf]

Reference List: Supplementary Table S1 and Supplementary Table S2

1. Andrews, G. (2016). How neuroscience informs teaching. In M. A. Maddix & D. G. Blevins (Eds.), *Neuroscience and Christian Formation.* (pp. 21–32). IAP Information Age Publishing.
2. Bakar, M. A. A., & Ghani, A. T. A. (2022). The impact of neuroscience literacy on sustainability of the students' mathematics learning environment. *Journal of Sustainability Science and Management*, *17*(9), 148–161. https://doi.org/10.46754/jssm.2022.09.010
3. Bamber, R. (2019). How can neuroscience inform our coaching practice—Six strategies to facilitate an optimal brain state in coaches. *Development and Learning in Organizations*, *33*(4), 13–15. https://doi.org/10.1108/DLO-01-2019-0007
4. Beeson, E. T., & Field, T. A. (2017). Neurocounseling: A New Section of the Journal of Mental Health Counseling. *Journal of Mental Health Counseling*, *39*(1), 71–83. https://doi.org/10.17744/mehc.39.1.06
5. Beeson, E. T., & Miller, R. M. (2019). Grounding Neuro-Informed Practice in a Humanistic Framework: A Response to Wilkinson. *Journal of Humanistic Counseling*, *58*(2), 95–107. https://doi.org/10.1002/johc.12099
6. Billington, T. (2017). Educational inclusion and critical neuroscience: Friends or foes? *International Journal of Inclusive Education*, *21*(8), 866–880. https://doi.org/10.1080/13603116.2017.1283717
7. Blackburn, C. A. S. (2009). *The effect of brain-based instructional techniques on the reading skills of elementary students* (Publication No. 3359879) [Doctoral dissertation, Walden University]. ProQuest Dissertations and Theses.
8. Brewer, W., & Murphy, B. (2021). Neuro-informed psychotherapy, a manualised intervention

for accessing marginalised youth: Clinical rationale, theoretical underpinnings and practice. In A. R. Yung, J. Cotter & P. D. McGarry (Eds.), *Youth mental health: Approaches to emerging mental ill-health in young people* (pp. 69–86). Routledge. https://doi.org/10.4324/9780429285806-7

9. Busso, D. S., & Pollack, C. (2015). No brain left behind: Consequences of neuroscience discourse for education. *Learning, Media and Technology*, *40*(2), 168–186. https://doi.org/10.1080/17439884.2014.908908
10. Buzick, J. (2020). *Understanding the biopsychological effects of trauma on learning: An investigation of interventions to support faculty* (Publication No. 27669697) [Doctoral dissertation, Seton Hall University]. ProQuest Dissertations and Theses.
11. Caine, R. N. (2008). How neuroscience informs our teaching of elementary students. In C. C. Block & S. R. Parris (Eds.), *Comprehension instruction: Research-based best practices* (pp. 127–141). The Guilford Press.
12. Camargo, A., Hossain, E., Aliko, S., Akinola-Odusola, D., & Artus, J. (2020). Neuroscience, urban regeneration and urban health. *Journal of Urban Regeneration and Renewal*, *13*(3), 280–289.
13. Center on the Developing Child at Harvard University. (2010). *The Foundations of Lifelong Health Are Built in Early Childhood.* https://harvardcenter.wpenginepowered.com/wp-content/uploads/2010/05/Foundations-of-Lifelong-Health.pdf
14. Center on the Developing Child at Harvard University. (2016a). *Applying the Science of Child Development in Child Welfare Systems*. https://harvardcenter.wpenginepowered.com/wp-content/uploads/2016/10/HCDC_ChildWelfareSystems_rev2017.pdf
15. Center on the Developing Child at Harvard University. (2016b). *From Best Practices to*

Breakthrough Impacts: A Science-Based Approach to Building a More Promising Future for Young Children and Families. https://harvardcenter.wpenginepowered.com/wp-content/uploads/2016/05/From_Best_Practices_to_Breakthrough_Impacts-4.pdf

16. Chang, Z., Schwartz, M. S., Hinesley, V., & Dubinsky, J. M. (2021). Neuroscience Concepts Changed Teachers' Views of Pedagogy and Students. *Frontiers in Psychology*, *12*. https://doi.org/10.3389/fpsyg.2021.685856
17. Chapko, D. (2015). *Early Childhood Development and Skills across the Life-Course through the Lens of the Developing Brain*. Health. Nutrition and Population Discussion Papers. https://hdl.handle.net/10986/24382
18. Clement, N. D., & Lovat, T. (2012). Neuroscience and Education: Issues and Challenges for Curriculum. *Curriculum Inquiry*, *42*(4), 534–557. https://doi.org/10.1111/j.1467-873X.2012.00602.x
19. Connell, M. W. (2006). *Foundations of educational neuroscience: Integrating theory, experiment, and design* (Publication No. 3207712) [Doctoral dissertation, Harvard University]. ProQuest Dissertation & Theses.
20. Cormier, S. R. (2021). *Exploring counselor educators' experiences with neuroscience-informed counseling pedagogy* (Publication No. 27964445) [Doctoral dissertation, University of Northern Colorado]. ProQuest Dissertation & Theses.
21. Cuartas, J., Baker-Henningham, H., Cepeda, A., Rey-Guerra, C., & Instituto Colombiano de Bienestar Familiar (ICBF) Early Childhood Team. (2022). The Apapacho Violence Prevention Parenting Program: Conceptual Foundations and Pathways to Scale. *International Journal of Environmental Research and Public Health*, *19*(14). https://doi.org/10.3390/ijerph19148582

22. De Bellis, M. D., & Zisk, A. (2014). The Biological Effects of Childhood Trauma. *Child and Adolescent Psychiatric Clinics of North America*, *23*(2), 185–222. https://doi.org/10.1016/j.chc.2014.01.002

23. de Kogel, C. H., Schrama, W. M., & Smit, M. (2014). Civil Law and Neuroscience. *Psychiatry, Psychology and Law*, *21*(2), 272–285. https://doi.org/10.1080/13218719.2013.808978

24. de Nooyer, K. M., & Lingard, M. W. (2017). Applying principles of the Neurosequential Model of Therapeutics across an adolescent day program and inpatient unit. *Australasian Psychiatry*, *25*(2), 150–153. https://doi.org/10.1177/1039856216658824

25. Deans, C., & Larsen, E. (2022). Brain-based learning: Beliefs and practice in one Australian primary school implementing a neuroscience pedagogical framework. *Australian Journal of Teacher Education*, *47*(10), 18–38. https://doi.org/10.14221/ajte.2022v47n10.2

26. Di Gesu, F. (2014). The NE.FO.DO project: A neurodidactic of foreign language. *Proceedings of the 6th International Conference on Education and New Learning Technologies, 2,* 4671–4676.

27. DiPietro, J. A. (2000). Baby and the brain: Advances in child development. *Annual Review of Public Health, 21,* 455-471. https://doi.org/10.1146/annurev.publhealth.21.1.455

28. Dow, M. (2008). Buddhism, psychology, and neuroscience: The promises and pitfalls of a neurobiologically informed contemplative psychotherapy. In F. J. Kaklauskas, S. Nimanheminda, L. Hoffman, M. S. Jack & J. Perlstein (Eds.), *Brilliant sanity: Buddhist approaches to psychotherapy* (pp. 99–129). University of the Rockies Press.

29. Dubinsky, J. M. (2010). Neuroscience education for prekindergarten-12 teachers. *Journal of Neuroscience*, *30*(24), 8057–8060. https://doi.org/10.1523/JNEUROSCI.2322-10.2010

30. Edelenbosch, R., Kupper, F., Krabbendam, L., & Broerse, J. E. W. (2015). Brain-based learning

and educational neuroscience: Boundary work. *Mind, Brain, and Education*, *9*(1), 40–49. https://doi.org/10.1111/mbe.12066

31. Ekhtiari, H., Rezapour, T., Aupperle, R. L., & Paulus, M. P. (2017). Neuroscience-informed psychoeducation for addiction medicine: A neurocognitive perspective. *Progress in Brain Research*, *235*, 239-264. https://doi.org/10.1016/bs.pbr.2017.08.013
32. Engle, P., & Huffman, S. L. (2010). Growing children's bodies and minds: Maximizing child nutrition and development. *Food and Nutrition Bulletin*, *31*, S186–S197. https://doi.org/10.1177/15648265100312s211
33. Farah, M. J. (2018). Socioeconomic status and the brain: Prospects for neuroscience-informed policy. *Nature Reviews Neuroscience*, *19*(7), 428–438. https://doi.org/10.1038/s41583-018-0023-2
34. Farrugia, A., & Fraser, S. (2017). Young brains at risk: Co-constituting youth and addiction in neuroscience-informed Australian drug education. *BioSocieties*, *12*(4), 588–610. https://doi.org/10.1057/s41292-017-0047-2
35. Field, T. A., Beeson, E. T., & Jones, L. K. (2015). The New ABCs: A Practitioner's Guide to Neuroscience-Informed Cognitive-Behavior Therapy. *Journal of Mental Health Counseling*, *37*(3), 206–220. https://doi.org/10.17744/1040-2861-37.3.206
36. Field, T. A., Beeson, E. T., & Jones, L. K. (2016). Neuroscience-Informed Cognitive-Behavior Therapy in Clinical Practice: A Preliminary Study. *Journal of Mental Health Counseling*, *38*(2), 139–154. https://doi.org/10.17744/mehc.38.2.05
37. Field, T. A., Beeson, E. T., Jones, L. K., & Miller, R. (2017). Counselor Allegiance and Client Expectancy in Neuroscience-Informed Cognitive-Behavior Therapy: A 12-Month Qualitative Follow-Up. *Journal of Mental Health Counseling*, *39*(4), 351–365.

https://doi.org/10.17744/mehc.39.4.06

38. Fishbane, M. D. (2016). The neurobiology of relationships. In T. L. Sexton & J. Lebow (Eds.), *Handbook of family therapy* (pp. 48–65). Routledge.

39. Fisher, M., Herman, A., Stephens, D. B., & Vinogradov, S. (2016). Neuroscience-informed computer-assisted cognitive training in schizophrenia. *Annals of the New York Academy of Sciences*, *1366*(1), 90-114. https://doi.org/10.1111/nyas.13042

40. Ford, J. (2015). An affective cognitive neuroscience-based approach to PTSD psychotherapy: The TARGET model. *Journal of Cognitive Psychotherapy*, *29*(1), 68–91. https://doi.org/10.1891/0889-8391.29.1.68

41. Frederickson, N., Jones, A. P., Warren, L., Deakes, T., & Allen, G. (2013). Can developmental cognitive neuroscience inform intervention for social, emotional and behavioural difficulties (SEBD)? *Emotional and Behavioural Difficulties*, *18*(2), 135–154. https://doi.org/10.1080/13632752.2012.757097

42. Friedman, D. (2006). *Interaction and the Architecture of the Brain*. National Scientific Council on the Developing Child. https://www.overcominghateportal.org/uploads/5/4/1/5/5415260/social_brain_nscdc.pdf

43. Geake, J. (2011). Position Statement on Motivations, Methodologies, and Practical Implications of Educational Neuroscience Research: fMRI Studies of the Neural Correlates of Creative Intelligence. In K. E. Patten & S. R. Campbell (Eds.), *Educational Neuroscience* (pp. 42–46). Wiley-Blackwell. https://doi.org/10.1002/9781444345827.ch7

44. Goodwin, B. (2018). *Student Learning That Works: How Brain Science Informs a Student Learning Model* (ED587406). ERIC. https://eric.ed.gov/?id=ED587406

45. Greenberg, M. T., Kusché, C. A., & Riggs, N. (2004). The PATHS Curriculum: Theory and

Research on Neurocognitive Development and School Success. In J. E. Zins, R. P. Weissberg, M. C. Wang & H. J. Walberg (Eds.), *Building academic success on social and emotional learning: What does the research say?* (pp. 170–188). Teachers College Press.

46. Gunnar, M. R. (2006). *Toxic Stress: Implications for Policy & Practice. An Interview with Developmental Psychologist Megan R. Gunnar* (ED503055). ERIC. https://eric.ed.gov/?id=ED503055
47. Hardiman, M. (2012). Informing Pedagogy Through the Brain-Targeted Teaching Model. *Journal of Microbiology & Biology Education*, *13*(1), 11–16. https://doi.org/10.1128/jmbe.v13i1.354
48. Hardiman, M., Rinne, L., Gregory, E., & Yarmolinskaya, J. (2012). Neuroethics, Neuroeducation, and Classroom Teaching: Where the Brain Sciences Meet Pedagogy. *Neuroethics*, *5*(2), 135–143. https://doi.org/10.1007/s12152-011-9116-6
49. Hohnen, B. (2017). Promoting educational success: How findings from neuroscience can guide educators to work optimally with the brain. In D. Skuse, H. Bruce & L. Dowdney (Eds.), *Child psychology and psychiatry: Frameworks for clinical training and practice* (pp. 413–419). Wiley-Blackwell. https://doi.org/10.1002/9781119170235.ch46
50. Husak, D., & Murphy, E. (2013). The relevance of the neuroscience of addiction to criminal law. In S. J. Morse & A. L. Roskies (Eds.), *A primer on criminal law and neuroscience: A contribution of the Law and Neuroscience Project, supported by the MacArthur Foundation* (pp. 216–239). Oxford University Press. https://doi.org/10.1093/acprof:oso/9780199859177.003.0008
51. Iacona, J., & Johnson, S. (2018). Neurobiology of Trauma and Mindfulness for Children. *Journal of Trauma Nursing*, *25*(3), 187–191.

https://doi.org/10.1097/JTN.0000000000000365

52. Iyengar, V., Ghosh, D., Smith, T., & Krueger, F. (2021). Age-Related Changes in Interpersonal Trust Behavior: Can Neuroscience Inform Public Policy? *NAM Perspectives*, *2019*. https://doi.org/10.31478/201906c
53. JohnBull, R. M., & Hardiman, M. M. (2023). Exploring Changes in Teacher Self-Efficacy Through Neuroeducation Professional Development. *The Teacher Educator*, *59*(2), 175-195. https://doi.org/10.1080/08878730.2023.2214555
54. Kim, S. R., & Zalaquett, C. (2019). An exploratory study of prevalence and predictors of neuromyths among potential mental health counselors. *Journal of Mental Health Counseling*, *41*(2), 173–187. https://doi.org/10.17744/mehc.41.2.06
55. King, J. L., Kaimal, G., Konopka, L., Belkofer, C., & Strang, C. E. (2019). Practical Applications of Neuroscience-Informed Art Therapy. *Art Therapy*, *36*(3), 149–156. https://doi.org/10.1080/07421656.2019.1649549
56. Liu, S., & Fisher, P. A. (2022). Early experience unpredictability in child development as a model for understanding the impact of the COVID-19 pandemic: A translational neuroscience perspective. *Developmental Cognitive Neuroscience*, *54*. https://doi.org/10.1016/j.dcn.2022.101091
57. Logue, M. E. (2000). *Implications of Brain Development Research for Even Start Family Literacy Programs. Look at Even Start* (ED446832). ERIC. https://eric.ed.gov/?id=ED446832
58. Long, D. A., Waak, M., Doherty, N. N., & Dow, B. L. (2022). Brain-Directed Care: Why Neuroscience Principles Direct PICU Management beyond the ABCs. *Children*, *9*(12). https://doi.org/10.3390/children9121938

59. Lowe, P., Lee, E., & Macvarish, J. (2015). Biologising parenting: Neuroscience discourse, English social and public health policy and understandings of the child. *Sociology of Health & Illness*, *37*(2), 198–211. https://doi.org/10.1111/1467-9566.12223

60. Lown, B. A. (2016). A social neuroscience-informed model for teaching and practising compassion in health care. *Medical Education*, *50*(3), 332–342. https://doi.org/10.1111/medu.12926

61. Lucero, I. (2018). Written in the Body?: Healing the Epigenetic Molecular Wounds of Complex Trauma Through Empathy and Kindness. *Journal of Child and Adolescent Trauma*, *11*(4), 443–455. https://doi.org/10.1007/s40653-018-0205-0

62. Luke, C. (2019). Response to Wilkinson: A Neuro-Informed Humanistic Perspective. *Journal of Humanistic Counseling*, *58*(2), 86–94. https://doi.org/10.1002/johc.12098

63. Luke, C., Beeson, E. T., Miller, R., Field, T. A., & Jones, L. K. (2020). Counselors' perceptions of ethical considerations for integrating neuroscience with counseling. *The Professional Counselor*, *10*(2), 204–219. https://doi.org/10.15241/cl.10.2.204

64. Mason, C., Kelly, B. L., & McConchie, V. (2020). Including Neuroscience in Social Work Education: Introducing Graduate Students to the Neurosequential Model of Therapeutics. *Journal of Teaching in Social Work*, *40*(4), 352–371. https://doi.org/10.1080/08841233.2020.1788692

65. Matsumoto, Y., Ishimoto, Y., & Takizawa, Y. (2020). Examination of the effectiveness of Neuroscience-Informed Child Education (NICE) within Japanese school settings. *Children and Youth Services Review*, *118*. https://doi.org/10.1016/j.childyouth.2020.105405

66. McEwen, B. S. (2017). Integrative medicine: Breaking down silos of knowledge and practice an epigenetic approach. *Metabolism: Clinical and Experimental*, *69*, S21–S29.

https://doi.org/10.1016/j.metabol.2017.01.018

67. Medalia, A., & Bellucci, D. (2012). Neuropsychologically informed interventions to treat cognitive impairment in schizophrenia. In B. A. Marcopulos & M. M. Kurtz (Eds.), *Clinical neuropsychological foundations of schizophrenia* (pp. 275–301). Psychology Press.
68. Millei, Z., & Joronen, M. (2016). The (bio)politicization of neuroscience in Australian early years policies: Fostering brain-resources as human capital. *Journal of Education Policy*, *31*(4), 389–404. https://doi.org/10.1080/02680939.2016.1148780
69. Miller, R. (2016). Neuroeducation: Integrating brain-based psychoeducation into clinical practice. *Journal of Mental Health Counseling*, *38*(2), 103–115. https://doi.org/10.17744/mehc.38.2.02
70. Moore, K. S., & Lagasse, A. B. (2018). Parallels and divergence between neuroscience and humanism: Considerations for the music therapist. *Music Therapy Perspectives*, *36*(2), 267–276. https://doi.org/10.1093/mtp/miy011
71. National Scientific Council on the Developing Child. (2004a). *Young Children Develop in an Environment of Relationships: Working Paper No. 1.* https://developingchild.harvard.edu/wp-content/uploads/2004/04/Young-Children-Develop-in-an-Environment-of-Relationships.pdf
72. National Scientific Council on the Developing Child. (2004b). *Children's Emotional Development Is Built into the Architecture of Their Brains: Working Paper No. 2*. https://developingchild.harvard.edu/wp-content/uploads/2004/04/Childrens-Emotional-Development-Is-Built-into-the-Architecture-of-Their-Brains.pdf
73. National Scientific Council on the Developing Child. (2005). *Excessive Stress Disrupts the Architecture of the Developing Brain: Working Paper No. 3*.

https://edn.ne.gov/cms/sites/default/files/u1/pdf/se05SE2%20Stress%20Disrupts%20Architecture%20Dev%20Brain%203.pdf

74. National Scientific Council on the Developing Child. (2007a). *The Science of Early Childhood Development: Closing the Gap between What We Know and What We Do.* https://developingchild.harvard.edu/wp-content/uploads/2015/05/Science_Early_Childhood_Development.pdf
75. National Scientific Council on the Developing Child. (2007b). *The Timing and Quality of Early Experiences Combine to Shape Brain Architecture. Working Paper No. 5.* https://harvardcenter.wpenginepowered.com/wp-content/uploads/2007/05/Timing_Quality_Early_Experiences-1.pdf
76. National Scientific Council on the Developing Child. (2014). *A Decade of Science Informing Policy: The Story of the National Scientific Council on the Developing Child.* https://developingchild.harvard.edu/wp-content/uploads/2015/09/A-Decade-of-Science-Informing-Policy.pdf
77. Navalta, C. P., McGee, L., & Underwood, J. (2018). Adverse Childhood Experiences, Brain Development, and Mental Health: A Call for Neurocounseling. *Journal of Mental Health Counseling*, *40*(3), 266–278. https://doi.org/10.17744/mehc.40.3.07
78. Noble, D. J., Martin, K., Qin, L., Britto, P., O'sullivan, M., Popkins, J., Pouwels, R., Scherpbier, R. W., & Flowers, R. (2017). What could cognitive capital mean for China's children? *PsyCh Journal*, *6*(2), 153–160. https://doi.org/10.1002/pchj.170
79. OECD. (2007). *Understanding the brain: The birth of a learning science.* https://doi.org/10.1787/9789264029132-en
80. Parris, S. R. (2008). How neuroscience informs our teaching of adolescent students. In C. C.

Block & S. R. Parris (Eds.), *Comprehension instruction: Research-based best practices* (pp. 142–155). The Guilford Press.

81. Perry, B. D. (2014). The Neurosequential Model of Therapeutics: Application of a developmentally sensitive and neurobiology-informed approach to clinical problem solving in maltreated children. In K. Brandt, B. D. Perry, S. Seligman & E. Tronick (Eds.), *Infant and early childhood mental health: Core concepts and clinical practice* (pp. 21–53). American Psychiatric Publishing.
82. Perry, B. D., & Dobson, C. L. (2013). The neurosequential model of therapeutics. In J. D. Ford & C. A. Courtois (Eds.), *Treating complex traumatic stress disorders in children and adolescents: Scientific foundations and therapeutic models* (pp. 249–260). The Guilford Press.
83. Petrocchi, N., Di Bello, M., Cheli, S., & Ottaviani, C. (2022). Compassion focused therapy and the body: How physiological underpinnings of prosociality inform clinical practice. In P. Gilbert & G. Simos (Eds.), *Compassion Focused Therapy: Clinical Practice and Applications* (pp. 345–359). Routledge. https://doi.org/10.4324/9781003035879-12
84. Prendiville, E., & Howard, J. (2017). Neurobiologically informed psychotherapy. In E. Prendiville & J. Howard (Eds.), *Creative psychotherapy: Applying the principles of neurobiology to play and expressive arts-based practice* (pp. 21–37). Routledge.
85. Pykett, J. (2019). A critical neuro-geography of behaviourally and neuroscientifically informed public policy. In A. Whitworth (Ed.), *Towards a Spatial Social Policy: Bridging the Gap Between Geography and Social Policy* (pp. 127–145). Policy Press.
86. Rauch, S. A. M., & McLean, C. P. (2021). In vivo exposure and neuroscience. In S. A. M. Rauch & C. P. McLean (Eds.), *Retraining the brain: Applied neuroscience in exposure therapy for*

PTSD (pp. 109–114). American Psychological Association. https://doi.org/10.1037/0000242-009

87. Ray, L. A., Nieto, S. J., & Grodin, E. N. (2023). Translational models of addiction phenotypes to advance addiction pharmacotherapy. *Annals of the New York Academy of Sciences, 1519*(1), 118-128. https://doi.org/10.1111/nyas.14929
88. Rezapour, T., Assari, S., Kirlic, N., Vassileva, J., & Ekhtiari, H. (2021). Enhancing cognitive resilience in adolescence and young adults: A multidimensional approach. In J. M. Croff & J. Beaman (Eds.), *Family resilience and recovery from opioids and other addictions* (pp. 45–64). Springer. https://doi.org/10.1007/978-3-030-56958-7_3
89. Rezapour, T., Aupperle, R. L., Paulus, M. P., & Ekhtiari, H. (2020). Clinical translation and implementation neuroscience for novel cognitive interventions in addiction medicine. In A. Verdejo-Garcia (Ed.), *Cognition and Addiction: A Researcher's Guide from Mechanisms Towards Interventions* (pp. 393–404). https://doi.org/10.1016/B978-0-12-815298-0.00029-0
90. Rezapour, T., Barzegari, M., Sharifi, E., Malmir, N., Ghiasvand, H., Salehi, M., Noroozi, A., & Ekhtiari, H. (2021). Neuroscience-Informed Psychoeducation for Recovery: A Program to Promote Metacognition in People With Substance Use Disorders. *Basic and Clinical Neuroscience*, *12*(5), 597–606. https://doi.org/10.32598/bcn.2021.809.3
91. Russell-Chapin, L. A. (2016). Integrating Neurocounseling into the Counseling Profession: An Introduction. *Journal of Mental Health Counseling*, *38*(2), 93–102. https://doi.org/10.l7744/mehc.38.2.01
92. Russell-Chapin, L. A., Field, T. A., & Jones, L. K. (2017). Ten practical guidelines for neurocounseling. In T. A. Field, L. K. Jones & L. A. Russell-Chapin (Eds.), *Neurocounseling: Brain-based clinical approaches* (pp. 227–235). American Counseling

Association.

93. Sanabria, S. (2020). Neuro-informed college counseling. In D. A. Paladino, L. M. Gonzalez & J. C. Watson (Eds.), *College counseling and student development: Theory, practice, and campus collaboration* (pp. 201–216). American Counseling Association.
94. Schildkrout, B. (2016). How to move beyond the diagnostic and statistical manual of mental disorders/international classification of diseases. *Journal of Nervous and Mental Disease*, *204*(10), 723–727. https://doi.org/10.1097/NMD.0000000000000547
95. Schmidt, U., Oldershaw, A., & van Elburg, A. (2011). Translating experimental neuroscience into treatment of eating disorders: Two examples. *Current Topics in Behavioral Neurosciences*, *6*(1), 253-268. https://doi.org/10.1007/7854_2010_76
96. Shi, T., & Blau, E. (2020). Contemporary Theories of Learning and Pedagogical Approaches for All Students to Achieve Success. In Y. Inoue-Smith & T. McVey (Eds.), *Optimizing Higher Education Learning Through Activities and Assessments* (pp. 20–37). IGI Global. https://doi.org/10.4018/978-1-7998-4036-7.ch002
97. Shonkoff, J. (2011). Protecting Brains, Not Simply Stimulating Minds: Investing Early in Education. *Science*, *333*(6045), 982–983.
98. Shonkoff, J. (2012). Leveraging the biology of adversity to address the roots of disparities in health and development. *Proceedings of the National Academy of Sciences, 109*(Supplement 2), 17302–17307. https://doi.org/10.1073/pnas.1121259109
99. Shonkoff, J. (2014). Excessive stress disrupts the development of brain architecture: National Scientific Council on the Developing Child. *Journal of Children's Services*, *9*(2), 143–153. https://doi.org/10.1108/JCS-01-2014-0006
100. Shonkoff, J., & Bales, S. N. (2011). Science Does Not Speak for Itself: Translating Child

Development Research for the Public and Its Policymakers. *Child Development*, *82*(1), 17–32. https://doi.org/10.1111/j.1467-8624.2010.01538.x

101. Shonkoff, J., & Fisher, P. (2013). Rethinking evidence-based practice and two-generation programs to create the future of early childhood policy. *Development and Psychopathology*, *25*(4), 1635–1653. https://doi.org/10.1017/S0954579413000813

102. Shonkoff, J., & Garner, A. (2012). The Lifelong Effects of Early Childhood Adversity and Toxic Stress. *Pediatrics*, *129*(1), e232–e246. https://doi.org/10.1542/peds.2011-2663

103. Sigman, M., Peña, M., Goldin, A. P., & Ribeiro, S. (2014). Neuroscience and education: Prime time to build the bridge. *Nature Neuroscience*, *17*(4), 497–502. https://doi.org/10.1038/nn.3672

104. Sinnamon, G. C. B. (2019). Eight core principles of neurobiologically informed interventions for trauma from childhood maltreatment. In I. Bryce, Y. Robinson & W. Petherick (Eds.), *Child Abuse and Neglect: Forensic Issues in Evidence, Impact and Management* (pp. 343–370). Academic Press. https://doi.org/10.1016/B978-0-12-815344-4.00018-0

105. Skodje-Mack, B. K. (2022). *Navigating integration: A grounded theory of practicing clinicians' experiences integrating neuroscience in their mental health clinical practice* (Publication No. 28499454) [Doctoral dissertation, Minnesota State University]. ProQuest Dissertation & Theses.

106. Stojek, M. M., McSweeney, L. B., & Rauch, S. A. M. (2018). Neuroscience informed prolonged exposure practice: Increasing efficiency and efficacy through mechanisms. *Frontiers in Behavioral Neuroscience*, *12*. https://doi.org/10.3389/fnbeh.2018.00281

107. Takizawa, Y., Murray, J., Bambling, M., Matsumoto, Y., Ishimoto, Y., Yamane, T., & Edirippulige, S. (2022). Integrating neuroscientific knowledge into psychotherapy amongst

Japanese psychotherapists: Presence, benefits, needs and cultural barriers. *Asia Pacific Journal of Counselling and Psychotherapy*, *13*(1), 81–100. https://doi.org/10.1080/21507686.2022.2035783

108. Thomas, M. S. C., Ansari, D., & Knowland, V. C. P. (2019). Annual Research Review: Educational neuroscience: Progress and prospects. *Journal of Child Psychology and Psychiatry*, *60*(4), 477–492. https://doi.org/10.1111/jcpp.12973

109. Verdejo-Garcia, A., Garcia-Fernandez, G., & Dom, G. (2019). Cognition and addiction. *Dialogues in Clinical Neuroscience*, *21*(3), 281–290. https://doi.org/10.31887/DCNS.2019.21.3/gdom

110. Verdejo-Garcia, A., Lorenzetti, V., Manning, V., Piercy, H., Bruno, R., Hester, R., Pennington, D., Tolomeo, S., Arunogiri, S., Bates, M. E., Bowden-Jones, H., Campanella, S., Daughters, S. B., Kouimtsidis, C., Lubman, D. I., Meyerhoff, D. J., Ralph, A., Rezapour, T., Tavakoli, H., … Ekhtiari, H. (2019). A Roadmap for Integrating Neuroscience Into Addiction Treatment: A Consensus of the Neuroscience Interest Group of the International Society of Addiction Medicine. *Frontiers in Psychiatry*, *10*. https://doi.org/10.3389/fpsyt.2019.00877

111. Vinke, A. J. G. (2022). Advocating the need for neuro-informed working with intercountry adoptees. *Child Abuse and Neglect*, *130*. https://doi.org/10.1016/j.chiabu.2022.105599

112. Vinogradov, S., Elmaghraby, R., & Pientka, L. (2021). Neuroscience-informed cognitive training for psychotic spectrum illnesses. In C. A. Tamminga, E. I. Ivleva, U. Reininghaus & J. van Os (Eds.), *Psychotic disorders: Comprehensive conceptualization and treatments* (pp. 592–600). Oxford University Press.

113. Wakefield, S., & McPherson, P. (2021). How the Evolving State of Neuroscience Informs the Definition of Adulthood: A Psychiatrist's Perspective. *Journal of Pediatric*

Neuropsychology, *7*(4), 161–168. https://doi.org/10.1007/s40817-021-00116-8

114. Watts-English, T., Fortson, B. L., Gibler, N., Hooper, S. R., & De Bellis, M. D. (2006). The psychobiology of maltreatment in childhood. *Journal of Social Issues*, *62*(4), 717–736. https://doi.org/10.1111/j.1540-4560.2006.00484.x

115. Weatherston, D., & Rosenblum, K. L. (2018). Promoting early relationships in infancy and early parenthood: Integrating social and emotional policy, practice, and research. In A. S. Morris & A. C. Williamson (Eds.), *Building early social and emotional relationships with infants and toddlers: Integrating research and practice* (pp. 325–341). Springer. https://doi.org/10.1007/978-3-030-03110-7_14

116. Zaleski, K. L., Johnson, D. K., & Klein, J. T. (2016). Grounding Judith Herman's Trauma Theory within Interpersonal Neuroscience and Evidence-Based Practice Modalities for Trauma Treatment. *Smith College Studies in Social Work*, *86*(4), 377–393. https://doi.org/10.1080/00377317.2016.1222110
